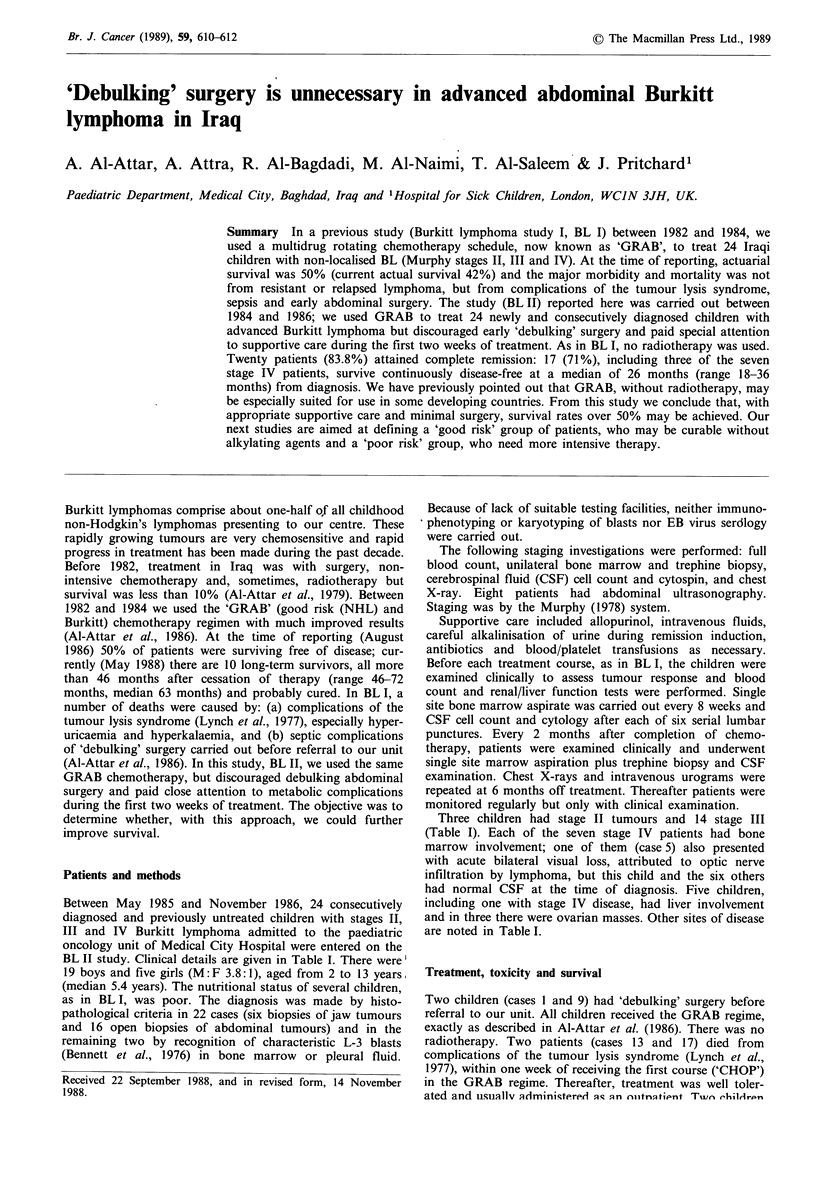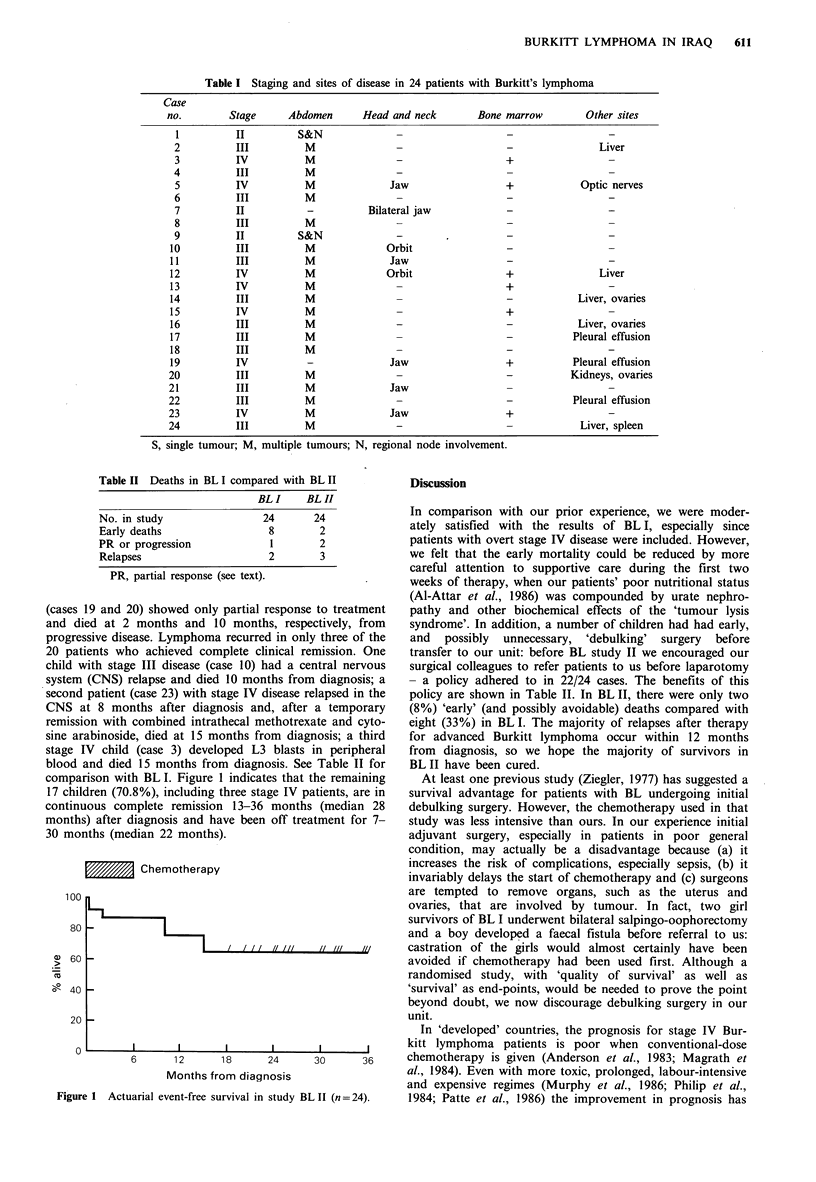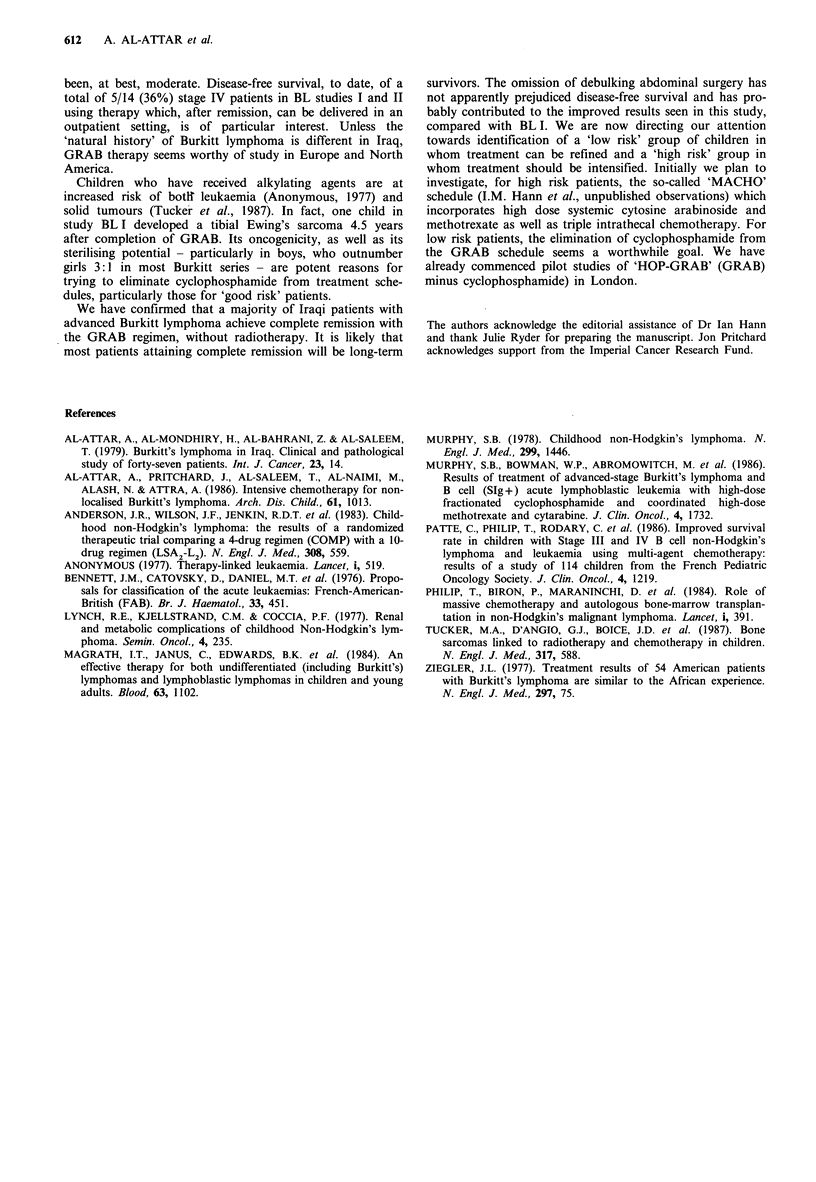# 'Debulking' surgery is unnecessary in advanced abdominal Burkitt lymphoma in Iraq.

**DOI:** 10.1038/bjc.1989.123

**Published:** 1989-04

**Authors:** A. al-Attar, A. Attra, R. al-Bagdadi, M. al-Naimi, T. al-Saleem, J. Pritchard

**Affiliations:** Paediatric Department, Medical City, Baghadad, Iraq.

## Abstract

In a previous study (Burkitt lymphoma study I, BL I) between 1982 and 1984, we used a multidrug rotating chemotherapy schedule, now known as 'GRAB', to treat 24 Iraqi children with non-localised BL (Murphy stages II, III and IV). At the time of reporting, actuarial survival was 50% (current actual survival 42%) and the major morbidity and mortality was not from resistant or relapsed lymphoma, but from complications of the tumour lysis syndrome, sepsis and early abdominal surgery. The study (BL II) reported here was carried out between 1984 and 1986; we used GRAB to treat 24 newly and consecutively diagnosed children with advanced Burkitt lymphoma but discouraged early 'debulking' surgery and paid special attention to supportive care during the first two weeks of treatment. As in BL I, no radiotherapy was used. Twenty patients (83.8%) attained complete remission: 17 (71%), including three of the seven stage IV patients, survive continuously disease-free at a median of 26 months (range 18-36 months) from diagnosis. We have previously pointed out that GRAB, without radiotherapy, may be especially suited for use in some developing countries. From this study we conclude that, with appropriate supportive care and minimal surgery, survival rates over 50% may be achieved. Our next studies are aimed at defining a 'good risk' group of patients, who may be curable without alkylating agents and a 'poor risk' group, who need more intensive therapy.


Br. J. Cancer (1989), 59, 610–612 © The Macmillan Press Ltd., 1989

### 'Debulking' surgery is unnecessary in advanced abdominal Burkitt lymphoma in Iraq

A. Al-Attar, A. Attra, R. Al-Bagdadi, M. Al-Naimi, T. Al-Saleem & J. Pritchard[1]

*Paediatric Department, Medical City, Baghdad, Iraq and [1]Hospital for Sick Children, London, WC1N 3JH, UK.*

**Summary** In a previous study (Burkitt lymphoma study I, BL I) between 1982 and 1984, we used a multidrug rotating chemotherapy schedule, now known as 'GRAB', to treat 24 Iraqi children with non-localised BL (Murphy stages II, III and IV). At the time of reporting, actuarial survival was 50% (current actual survival 42%) and the major morbidity and mortality was not from resistant or relapsed lymphoma, but from complications of the tumour lysis syndrome, sepsis and early abdominal surgery. The study (BL II) reported here was carried out between 1984 and 1986; we used GRAB to treat 24 newly and consecutively diagnosed children with advanced Burkitt lymphoma but discouraged early 'debulking' surgery and paid special attention to supportive care during the first two weeks of treatment. As in BL I, no radiotherapy was used. Twenty patients (83.8%) attained complete remission: 17 (71%), including three of the seven stage IV patients, survive continuously disease-free at a median of 26 months (range 18–36 months) from diagnosis. We have previously pointed out that GRAB, without radiotherapy, may be especially suited for use in some developing countries. From this study we conclude that, with appropriate supportive care and minimal surgery, survival rates over 50% may be achieved. Our next studies are aimed at defining a 'good risk' group of patients, who may be curable without alkylating agents and a 'poor risk' group, who need more intensive therapy.

Burkitt lymphomas comprise about one-half of all childhood non-Hodgkin's lymphomas presenting to our centre. These rapidly growing tumours are very chemosensitive and rapid progress in treatment has been made during the past decade. Before 1982, treatment in Iraq was with surgery, non-intensive chemotherapy and, sometimes, radiotherapy but survival was less than 10% (Al-Attar *et al.*, 1979). Between 1982 and 1984 we used the 'GRAB' (good risk (NHL) and Burkitt) chemotherapy regimen with much improved results (Al-Attar *et al.*, 1986). At the time of reporting (August 1986) 50% of patients were surviving free of disease; currently (May 1988) there are 10 long-term survivors, all more than 46 months after cessation of therapy (range 46–72 months, median 63 months) and probably cured. In BL I, a number of deaths were caused by: (a) complications of the tumour lysis syndrome (Lynch *et al.*, 1977), especially hyperuricaemia and hyperkalaemia, and (b) septic complications of 'debulking' surgery carried out before referral to our unit (Al-Attar *et al.*, 1986). In this study, BL II, we used the same GRAB chemotherapy, but discouraged debulking abdominal surgery and paid close attention to metabolic complications during the first two weeks of treatment. The objective was to determine whether, with this approach, we could further improve survival.

### Patients and methods

Between May 1985 and November 1986, 24 consecutively diagnosed and previously untreated children with stages II, III and IV Burkitt lymphoma admitted to the paediatric oncology unit of Medical City Hospital were entered on the BL II study. Clinical details are given in Table I. There were 19 boys and five girls (M:F 3.8:1), aged from 2 to 13 years (median 5.4 years). The nutritional status of several children, as in BL I, was poor. The diagnosis was made by histopathological criteria in 22 cases (six biopsies of jaw tumours and 16 open biopsies of abdominal tumours) and in the remaining two by recognition of characteristic L-3 blasts (Bennett *et al.*, 1976) in bone marrow or pleural fluid. Because of lack of suitable testing facilities, neither immunophenotyping or karyotyping of blasts nor EB virus serology were carried out.

The following staging investigations were performed: full blood count, unilateral bone marrow and trephine biopsy, cerebrospinal fluid (CSF) cell count and cytospin, and chest X-ray. Eight patients had abdominal ultrasonography. Staging was by the Murphy (1978) system.

Supportive care included allopurinol, intravenous fluids, careful alkalinisation of urine during remission induction, antibiotics and blood/platelet transfusions as necessary. Before each treatment course, as in BL I, the children were examined clinically to assess tumour response and blood count and renal/liver function tests were performed. Single site bone marrow aspirate was carried out every 8 weeks and CSF cell count and cytology after each of six serial lumbar punctures. Every 2 months after completion of chemotherapy, patients were examined clinically and underwent single site marrow aspiration plus trephine biopsy and CSF examination. Chest X-rays and intravenous urograms were repeated at 6 months off treatment. Thereafter patients were monitored regularly but only with clinical examination.

Three children had stage II tumours and 14 stage III (Table I). Each of the seven stage IV patients had bone marrow involvement; one of them (case 5) also presented with acute bilateral visual loss, attributed to optic nerve infiltration by lymphoma, but this child and the six others had normal CSF at the time of diagnosis. Five children, including one with stage IV disease, had liver involvement and in three there were ovarian masses. Other sites of disease are noted in Table I.

### Treatment, toxicity and survival

Two children (cases 1 and 9) had 'debulking' surgery before referral to our unit. All children received the GRAB regime, exactly as described in Al-Attar *et al.* (1986). There was no radiotherapy. Two patients (cases 13 and 17) died from complications of the tumour lysis syndrome (Lynch *et al.*, 1977), within one week of receiving the first course ('CHOP') in the GRAB regime. Thereafter, treatment was well tolerated and usually administered as an outpatient. Two children

Received 22 September 1988, and in revised form, 14 November 1988.

**Table I** Staging and sites of disease in 24 patients with Burkitt's lymphoma

| *Case no.* | *Stage* | *Abdomen* | *Head and neck* | *Bone marrow* | *Other sites* |
|---|---|---|---|---|---|
| 1 | II | S&N | – | – | – |
| 2 | III | M | – | – | Liver |
| 3 | IV | M | – | + | – |
| 4 | III | M | – | – | – |
| 5 | IV | M | Jaw | + | Optic nerves |
| 6 | III | M | – | – | – |
| 7 | II | – | Bilateral jaw | – | – |
| 8 | III | M | – | – | – |
| 9 | II | S&N | – | – | – |
| 10 | III | M | Orbit | – | – |
| 11 | III | M | Jaw | – | – |
| 12 | IV | M | Orbit | + | Liver |
| 13 | IV | M | – | + | – |
| 14 | III | M | – | – | Liver, ovaries |
| 15 | IV | M | – | + | – |
| 16 | III | M | – | – | Liver, ovaries |
| 17 | III | M | – | – | Pleural effusion |
| 18 | III | M | – | – | – |
| 19 | IV | – | Jaw | + | Pleural effusion |
| 20 | III | M | – | – | Kidneys, ovaries |
| 21 | III | M | Jaw | – | – |
| 22 | III | M | – | – | Pleural effusion |
| 23 | IV | M | Jaw | + | – |
| 24 | III | M | – | – | Liver, spleen |

S, single tumour; M, multiple tumours; N, regional node involvement.

**Table II** Deaths in BL I compared with BL II

| | *BL I* | *BL II* |
|---|---|---|
| No. in study | 24 | 24 |
| Early deaths | 8 | 2 |
| PR or progression | 1 | 2 |
| Relapses | 2 | 3 |

PR, partial response (see text).

(cases 19 and 20) showed only partial response to treatment and died at 2 months and 10 months, respectively, from progressive disease. Lymphoma recurred in only three of the 20 patients who achieved complete clinical remission. One child with stage III disease (case 10) had a central nervous system (CNS) relapse and died 10 months from diagnosis; a second patient (case 23) with stage IV disease relapsed in the CNS at 8 months after diagnosis and, after a temporary remission with combined intrathecal methotrexate and cytosine arabinoside, died at 15 months from diagnosis; a third stage IV child (case 3) developed L3 blasts in peripheral blood and died 15 months from diagnosis. See Table II for comparison with BL I. Figure 1 indicates that the remaining 17 children (70.8%), including three stage IV patients, are in continuous complete remission 13–36 months (median 28 months) after diagnosis and have been off treatment for 7–30 months (median 22 months).

**Figure 1** Actuarial event-free survival in study BL II ($n=24$).

### Discussion

In comparison with our prior experience, we were moderately satisfied with the results of BL I, especially since patients with overt stage IV disease were included. However, we felt that the early mortality could be reduced by more careful attention to supportive care during the first two weeks of therapy, when our patients' poor nutritional status (Al-Attar *et al.*, 1986) was compounded by urate nephropathy and other biochemical effects of the 'tumour lysis syndrome'. In addition, a number of children had had early, and possibly unnecessary, 'debulking' surgery before transfer to our unit: before BL study II we encouraged our surgical colleagues to refer patients to us before laparotomy – a policy adhered to in 22/24 cases. The benefits of this policy are shown in Table II. In BL II, there were only two (8%) 'early' (and possibly avoidable) deaths compared with eight (33%) in BL I. The majority of relapses after therapy for advanced Burkitt lymphoma occur within 12 months from diagnosis, so we hope the majority of survivors in BL II have been cured.

At least one previous study (Ziegler, 1977) has suggested a survival advantage for patients with BL undergoing initial debulking surgery. However, the chemotherapy used in that study was less intensive than ours. In our experience initial adjuvant surgery, especially in patients in poor general condition, may actually be a disadvantage because (a) it increases the risk of complications, especially sepsis, (b) it invariably delays the start of chemotherapy and (c) surgeons are tempted to remove organs, such as the uterus and ovaries, that are involved by tumour. In fact, two girl survivors of BL I underwent bilateral salpingo-oophorectomy and a boy developed a faecal fistula before referral to us: castration of the girls would almost certainly have been avoided if chemotherapy had been used first. Although a randomised study, with 'quality of survival' as well as 'survival' as end-points, would be needed to prove the point beyond doubt, we now discourage debulking surgery in our unit.

In 'developed' countries, the prognosis for stage IV Burkitt lymphoma patients is poor when conventional-dose chemotherapy is given (Anderson *et al.*, 1983; Magrath *et al.*, 1984). Even with more toxic, prolonged, labour-intensive and expensive regimes (Murphy *et al.*, 1986; Philip *et al.*, 1984; Patte *et al.*, 1986) the improvement in prognosis has been, at best, moderate. Disease-free survival, to date, of a total of 5/14 (36%) stage IV patients in BL studies I and II using therapy which, after remission, can be delivered in an outpatient setting, is of particular interest. Unless the 'natural history' of Burkitt lymphoma is different in Iraq, GRAB therapy seems worthy of study in Europe and North America.

Children who have received alkylating agents are at increased risk of both leukaemia (Anonymous, 1977) and solid tumours (Tucker et al., 1987). In fact, one child in study BL I developed a tibial Ewing's sarcoma 4.5 years after completion of GRAB. Its oncogenicity, as well as its sterilising potential – particularly in boys, who outnumber girls 3:1 in most Burkitt series – are potent reasons for trying to eliminate cyclophosphamide from treatment schedules, particularly those for 'good risk' patients.

We have confirmed that a majority of Iraqi patients with advanced Burkitt lymphoma achieve complete remission with the GRAB regimen, without radiotherapy. It is likely that most patients attaining complete remission will be long-term survivors. The omission of debulking abdominal surgery has not apparently prejudiced disease-free survival and has probably contributed to the improved results seen in this study, compared with BL I. We are now directing our attention towards identification of a 'low risk' group of children in whom treatment can be refined and a 'high risk' group in whom treatment should be intensified. Initially we plan to investigate, for high risk patients, the so-called 'MACHO' schedule (I.M. Hann et al., unpublished observations) which incorporates high dose systemic cytosine arabinoside and methotrexate as well as triple intrathecal chemotherapy. For low risk patients, the elimination of cyclophosphamide from the GRAB schedule seems a worthwhile goal. We have already commenced pilot studies of 'HOP-GRAB' (GRAB) minus cyclophosphamide) in London.

The authors acknowledge the editorial assistance of Dr Ian Hann and thank Julie Ryder for preparing the manuscript. Jon Pritchard acknowledges support from the Imperial Cancer Research Fund.

### References

AL-ATTAR, A., AL-MONDHIRY, H., AL-BAHRANI, Z. & AL-SALEEM, T. (1979). Burkitt's lymphoma in Iraq. Clinical and pathological study of forty-seven patients. *Int. J. Cancer*, **23**, 14.

AL-ATTAR, A., PRITCHARD, J., AL-SALEEM, T., AL-NAIMI, M., ALASH, N. & ATTRA, A. (1986). Intensive chemotherapy for non-localised Burkitt's lymphoma. *Arch. Dis. Child.*, **61**, 1013.

ANDERSON, J.R., WILSON, J.F., JENKIN, R.D.T. *et al.* (1983). Childhood non-Hodgkin's lymphoma: the results of a randomized therapeutic trial comparing a 4-drug regimen (COMP) with a 10-drug regimen ($LSA_2$-$L_2$). *N. Engl. J. Med.*, **308**, 559.

ANONYMOUS (1977). Therapy-linked leukaemia. *Lancet*, **i**, 519.

BENNETT, J.M., CATOVSKY, D., DANIEL, M.T. *et al.* (1976). Proposals for classification of the acute leukaemias: French-American-British (FAB). *Br. J. Haematol.*, **33**, 451.

LYNCH, R.E., KJELLSTRAND, C.M. & COCCIA, P.F. (1977). Renal and metabolic complications of childhood Non-Hodgkin's lymphoma. *Semin. Oncol.*, **4**, 235.

MAGRATH, I.T., JANUS, C., EDWARDS, B.K. *et al.* (1984). An effective therapy for both undifferentiated (including Burkitt's) lymphomas and lymphoblastic lymphomas in children and young adults. *Blood*, **63**, 1102.

MURPHY, S.B. (1978). Childhood non-Hodgkin's lymphoma. *N. Engl. J. Med.*, **299**, 1446.

MURPHY, S.B., BOWMAN, W.P., ABROMOWITCH, M. *et al.* (1986). Results of treatment of advanced-stage Burkitt's lymphoma and B cell (SIg+) acute lymphoblastic leukemia with high-dose fractionated cyclophosphamide and coordinated high-dose methotrexate and cytarabine. *J. Clin. Oncol.*, **4**, 1732.

PATTE, C., PHILIP, T., RODARY, C. *et al.* (1986). Improved survival rate in children with Stage III and IV B cell non-Hodgkin's lymphoma and leukaemia using multi-agent chemotherapy: results of a study of 114 children from the French Pediatric Oncology Society. *J. Clin. Oncol.*, **4**, 1219.

PHILIP, T., BIRON, P., MARANINCHI, D. *et al.* (1984). Role of massive chemotherapy and autologous bone-marrow transplantation in non-Hodgkin's malignant lymphoma. *Lancet*, **i**, 391.

TUCKER, M.A., D'ANGIO, G.J., BOICE, J.D. *et al.* (1987). Bone sarcomas linked to radiotherapy and chemotherapy in children. *N. Engl. J. Med.*, **317**, 588.

ZIEGLER, J.L. (1977). Treatment results of 54 American patients with Burkitt's lymphoma are similar to the African experience. *N. Engl. J. Med.*, **297**, 75.